# What Do Stroke Patients Look for in Game-Based Rehabilitation: A Survey Study

**DOI:** 10.1097/MD.0000000000003032

**Published:** 2016-03-18

**Authors:** Ya-Xuan Hung, Pei-Chen Huang, Kuan-Ta Chen, Woei-Chyn Chu

**Affiliations:** From the Department of Biomedical Engineering (Y-XH), National Yang-Ming University; Institute of Information Science (Y-XH), Academia Sinica; Rehabilitation Department (P-CH), Shin Kong Wu Ho-Su Memorial Hospital; Institute of Information Science (K-TC), Academia Sinica; and Department of Biomedical Engineering (W-CC), National Yang-Ming University, Taipei, Taiwan.

## Abstract

Stroke is one of the most common causes of physical disability, and early, intensive, and repetitive rehabilitation exercises are crucial to the recovery of stroke survivors. Unfortunately, research shows that only one third of stroke patients actually perform recommended exercises at home, because of the repetitive and mundane nature of conventional rehabilitation exercises. Thus, to motivate stroke survivors to engage in monotonous rehabilitation is a significant issue in the therapy process.

Game-based rehabilitation systems have the potential to encourage patients continuing rehabilitation exercises at home. However, these systems are still rarely adopted at patients’ places. Discovering and eliminating the obstacles in promoting game-based rehabilitation at home is therefore essential.

For this purpose, we conducted a study to collect and analyze the opinions and expectations of stroke patients and clinical therapists. The study is composed of 2 parts: Rehab-preference survey – interviews to both patients and therapists to understand the current practices, challenges, and expectations on game-based rehabilitation systems; and Rehab-compatibility survey – a gaming experiment with therapists to elaborate what commercial games are compatible with rehabilitation. The study is conducted with 30 outpatients with stroke and 19 occupational therapists from 2 rehabilitation centers in Taiwan.

Our surveys show that game-based rehabilitation systems can turn the rehabilitation exercises more appealing and provide personalized motivation for various stroke patients. Patients prefer to perform rehabilitation exercises with more diverse and fun games, and need cost-effective rehabilitation systems, which are often built on commodity hardware. Our study also sheds light on incorporating the existing design-for-fun games into rehabilitation system. We envision the results are helpful in developing a platform which enables rehab-compatible (i.e., existing, appropriately selected) games to be operated on commodity hardware and brings cost-effective rehabilitation systems to more and more patients’ home for long-term recovery.

## Abstract 2

Supplemental Digital Content is available in the text

## INTRODUCTION

Stroke constantly ranks 3rd among the leading causes of death and the most common cause of severe physical disability among adults in Taiwan.^1^ Moreover, about 800,000 Americans suffer from new or recurrent stoke each year,^2^ and the number is expected to grow as the population ages. Stroke causes various neurological impairments,^3^ including hemiparesis, motor incoordination, aphasia (loss of the ability of speech),^4^ apraxia (inability to execute learned purposeful movements),^5^ and perceptual disorders (hemispatial neglect). About 80% of stroke survivors suffer from significant motor impairments, require home cares, and face difficulty for returning to work.^6^ Recent study shows that intensive and repetitive rehabilitation exercises help stroke survivors recover from motor impairment.^7^

The capacity of rehabilitation centers, however, is way behind the number of stroke patients,^8,9^ forcing the therapists to give rehabilitation sessions with limited rehabilitation exercises. In order to achieve better performance and control, stroke patients are encouraged to perform most of daily rehabilitation exercises at home. Unfortunately, research shows that only 31% of stroke patients follow the therapists’ regimen,^10^ probably because of the repetitive nature of conventional rehabilitation exercises, and lack of supervision and feedbacks during exercises. Hence, engineers and researchers have looked into innovative technologies to better motivate stroke patients to continually perform rehabilitation exercises at home.

Interactive computer gaming technology has gained attention as components of rehabilitation. Built upon the recent advances in virtual reality (VR) and tracking devices, ranging from high-cost platform systems^11^ and robotics^12–14^ to low-cost off-the-shelf video gaming technologies (e.g., Nintendo Wii,^15^ Sony PlayStation 2 EyeToy,^16^ and Microsoft Kinect for Windows^17^) and tablet technology (e.g., iPad^18^ or Sony tablet Z^19^), game-based rehabilitation systems have been proposed to motivate patient during rehabilitation exercises. Generally, these systems work on the principle of providing the patients with an interactive user interface and implementing different task-oriented scenarios. With tracking device (hardware), users can do specific rehabilitation motions to interact with a graphic gaming interface (software). These gaming contents may be customized from scratch by researchers or selected from existing commercial games. Although these systems show great potential as an alternative to conventional therapy, current game-based rehabilitation systems still face unpopularity at home. There is a need for more in-depth understanding of the possible reasons for the barrier to implement these systems at home.

The overall goal of the current research is to develop a cost-effective game-based rehabilitation system and bring it into more patients’ home for long-term recovery. Firstly, the study explores the opinions of stroke patients and therapists – what do users want? We aim to find why they are not satisfied with the existing systems and identify the root causes. Secondly, we hypothesize that using existing, appropriately selected games will be a more efficient way to provide available games for rehabilitation. Therefore, to better understand the selection principles of rehab-compatible games, we propose a pilot survey to examine therapists’ opinions as they performed gaming activities on a multitouch tablet.

The rest of this paper is organized as follows. Section II gives a brief summary of technologies and games used in stroke rehabilitation systems. In Section III, the materials and proposed methodology are presented in detail. We present the results and discussions in Section IV and Section V, respectively. Finally, the conclusion is given in Section VI.

### Related Works

The related works reported in the paper can be classified into 2 major categories: game-based rehabilitation systems adopting with specific design-for-rehabilitation games and existing design-for-fun games.

### Rehabilitation Systems With Design-for-Rehabilitation Games

Several studies^20–23^ have focused on developing and testing systems designed specifically for stroke patients. Burke et al^21^ developed a series of webcam games for upper extremity stroke rehabilitation, which require low-cost webcams as well as a pair of colored gloves in order to operate. Alankus et al^20^ implemented 7 stroke rehabilitation games using both webcams and Wii remotes; more specifically, they used the accelerometers of Wii remotes attached to patients’ arms to detect motions for upper extremity exercises. Sin and Lee^24^ designed a study to investigate the effects of additional virtual reality training using Xbox Kinect and found that the stroke patients who received additional virtual reality training showed significantly improved function of the upper extremity. Ekbia et al^25^ designed an interactive therapy platform using Microsoft Kinect for Windows and evaluated usability and acceptability of the platform, involving 9 patients and 6 therapists. Both of participants reflected well on the rating components (appearance, graphics, game play, and overall appeal of the game). Annet et al^22^ developed a multitouch tabletop system under the guidance of occupational therapists (OTs). They compared the traditional exercises to the new tabletop-based exercises and found that more patients prefer the multitouch exercises than the traditional ones.

Although the aforementioned studies suggested the benefit of motivating patients with designed-for-rehabilitation games, it is worthy of note that these approaches are specific designed for 1 therapy (i.e., specific motions in particular joints), there is still lack of available games for rehabilitation probably due to many different requirements that have to be met.^26^

We also found that most of the games specifically designed for rehabilitation are similar with existing mobile games. Shah et al^27^ presented 3 different games, Marble Maze, Run Jack Run game, and Burger Hub game to achieve repetitive hand and wrist gestures, and applications (application softwares [APPs]) similar to these games can be easily found in Google Play and APP Store. The games of STROKE REHAB^28^ for patients with fine motor weakness is similar with Balloon Party.^29^ The dragging task and the tapping task in FINDEX^30^ (Android-based tablet games) may be imitating Pizza Maker^31^ and Piano Master.^32^ Some studies^9,33^ indicated that these customized games lack game design parameters, remain simplistic, and are only used to present the exercise tasks in a more visual and graphic manner. These games still have a long way to go in making progress in graphics, sound, game design, and, the most important factor, fun.

### Rehabilitation Systems With Existing Design-for-Fun Games

The effectiveness and the appropriateness of adopting commercial games on stroke rehabilitation were evaluated in some studies recently. Joo et al^34^ let 16 subjects with stroke took 6 sessions of upper limb exercises via a Nintendo Wii. The authors selected games from the Wii Sports software, such as boxing, bowling, and baseball. All subjects found Nintendo Wii gaming was enjoyable and appeared to be a feasible adjunctive device to augment conventional therapy. Paquin et al^35^ also investigated the effectiveness of using the Nintendo Wii as an intervention for fine motor recovery; the results show that commercial gaming can be a resource for chronic stroke survivors. Flynn et al^36^ conducted a survey to explore the attitudes of individuals with disabilities, using 4 off-the-shelf VR game-based systems (Sony PlayStation 2 EyeToy, Nintendo Wii, Novint Falcon,^37^ and an optical tracking System). Participants preferred EyeToy and Wii in overall preference and desired to use both of them frequently. Rand et al^38^ conducted a study to assess Rehab-lat (utilizing commercial game APPs selected by OTs) compared to conventional therapy. Potentially, Rehab-let might be ideal for individuals with mild stroke who are often not referred to formal rehabilitation.

To sum up, the evolving of game-based rehabilitation reveals a possible trend:

- more and more game-based rehabilitation systems are built using commodity hardware, such as tablets, Kinects, and Wii remotes
- most studies have focused on creating games from scratch, rather than adopting (already done and successful) designed-for-fun video games
- however, there is not enough games designed to be entertaining and motivational for the patients
- fortunately, commercial games selected by therapists may be a possible resource to enrich the game pool for rehabilitation use

Although the game-based rehabilitation systems have been proposed by many research groups and shown benefits on motivating patients. There are relatively few studies addressed the following 2 issues. First, most of the aforementioned studies conducted one-time studies and only focus on evaluating some specific systems. To the best of our knowledge, stroke patients and therapists’ opinions on these systems have not been rigorously studied in a longitudinal study. To truly understand these, the study presents the very 1st questionnaire survey of them on the existing game-based stroke rehabilitation systems. Second, the games which are customized by researchers and directly selected from existing commercial game pool still rely on therapists’ knowledge and experience. To date, however, no clear direction has been made to suggest how such knowledge and experience could be translated into well-defined rules of game recommendation. Thus, the other objective in our study is to identify which factors make existing commercial games compatible with rehabilitation use.

## METHODS

### Overview of Study Design

This study is consisted of 2 parts: rehab-preference survey and rehab-compatibility survey. In rehab-preference survey, since some visual and upper extremity impairments may prevent the stroke patients from filling out the questionnaires, interviews were conducted on individual patients with stroke (n = 30) and OTs (n = 19) by the 1st author, with the intention of understanding current game-based rehabilitation practices, the challenges faced, and their expectations on these systems. Each interview lasted for 30 to 40 minutes, and the answers were noted on survey sheets which were later quantitatively and qualitatively analyzed.

The purpose of rehab-compatibility survey is to see how therapists choose games which are compatible for rehabilitation use, and to make it a rule. A total of 24 game APPs used in the study (see Table 1) were downloaded from Google Play (Google, Mountain View, CA). Each game was preselected based on discussions with therapists who do not participant in the survey and was randomly evaluated by at least 5 OTs. Each experiment lasted about 1 hour, and 14 OTs were trained for playing 12 games (randomly selected from the game pool) on Sony Xperia Tablet Z^19^ with 10? multitouch LCD. Each game session lasted 3 minutes, OTs were asked to evaluate the commercial games’ features immediately following the completion of each session.

**TABLE 1:**
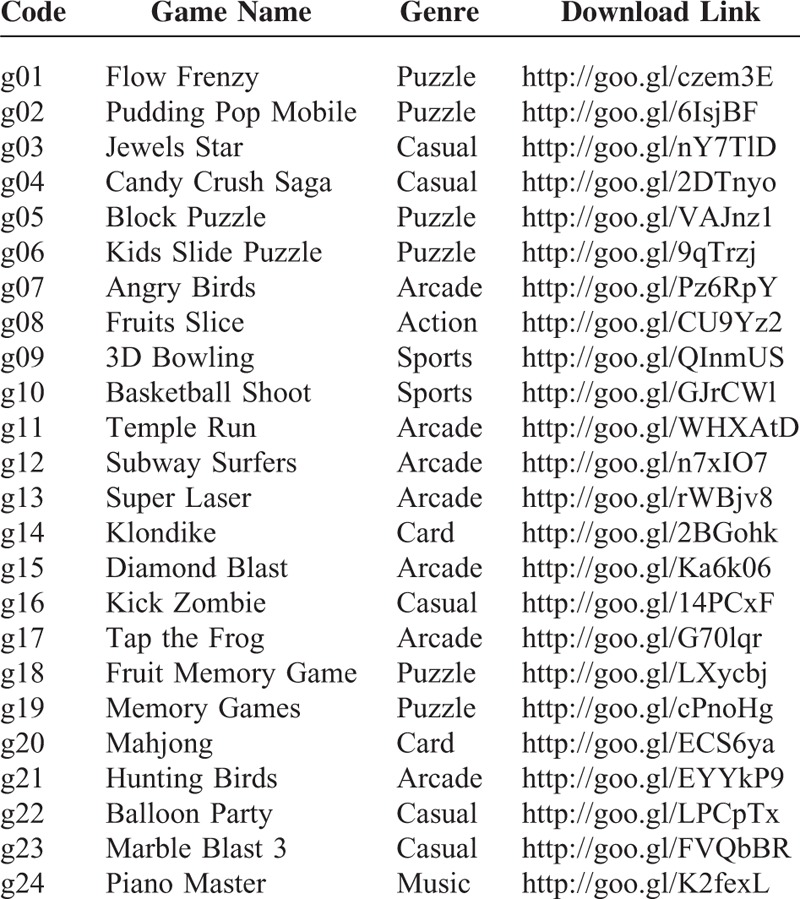
Game Used in the Study

### Survey Development and Instrument

- Rehab-preference survey for studying the opinions and expectation on game-based rehabilitation systems

The survey is conducted by a questionnaire (QN.I, see in supplementary document) for patients, which consists of 54 questions which are either multiple-choice or free-response questions. These questions are organized into 5 parts:

- Demographic information (12 questions), such as sex, age, education, and stroke nature.
- Current and past leisure activities (9 questions), such as favorite leisure activities and past gaming experience.
- Current rehabilitation status (16 questions), such as favorite rehabilitation equipment and opinions on performing rehabilitation exercises at home.
- Opinions on existing rehabilitation systems (4 questions), such as patients’ perception and incentive. The stroke patients were asked to provide opinions on the Reha-Slide system^39^ as it is the only game-based rehabilitation system adopted in the concerned rehabilitation center. And whether they would consider computer gaming as a part of their rehabilitation programme.
- Wish list for future rehabilitation systems (14 questions), such as desired hardware design factors and software functions.

The questionnaire for OTs consists of 3 parts, the part IV, part V from the patients’ questionnaire, and general comments on the game-based rehabilitation system.

Rehab-compatibility survey for identifying principles for rehab-compatible game selection

As patients have limited abilities due to stroke, the difficulty of games has been considered to play an important role in game compatibility. We designed the questionnaire (QN.II, see in supplementary document) consists of 8 rating components and is divided into 4 parts. The rating components were pilot tested with 2 therapists and refined to ensure clarity.

- Gaming operation including pace of games, average moving distance of 1 operation, and total moving range on tablet.
- Gaming interface including the size and number of game objects displayed on the screen.
- Gaming content, the degree on logical and conceptual challenges.
- Overall assessment including difficulty and compatibility level.

The questionnaire was conducted by 7-point Likert-scale and built with Google Forms (http://goo.gl/fduXyw). We list all rating components with its corresponding meaning in Table 2.

**TABLE 2:**
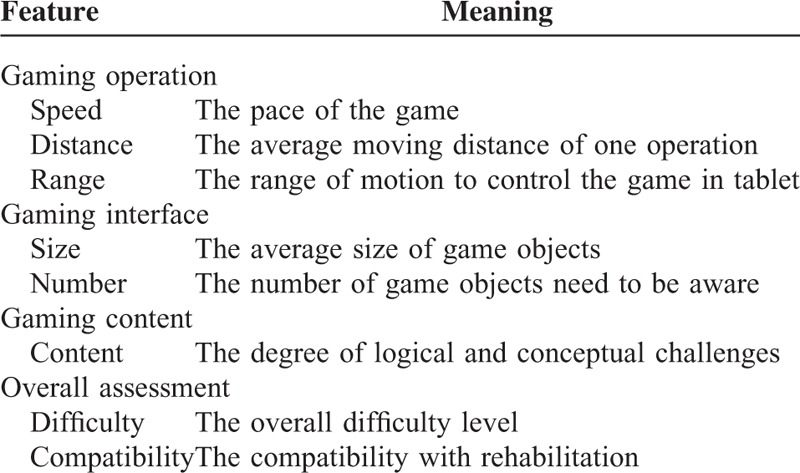
Rating Components

## PARTICIPANTS

### Patients With Stroke

A total of 30 outpatients with stroke were recruited in rehab-preference survey. These stroke patients were selected from the rehabilitation center of Shin Kong Wu Ho-Su Memorial Hospital by OTs. These patients did not have severe aphasia, cognitive impairment, visual impairment, or psychiatric illnesses, and thus were capable to participate in the survey. The study (approval number: AS-IRB02-103017) has been approved by the Institutional Review Board (IRB) on Biomedical Science Research of Academia Sinica. Informed consent was obtained from all individual participants included in the study.

The demographic and clinical characteristics of the stroke patients are given in Table 3. There are 22 male and 8 female patients, with mean (SD) age of 55 (18) years old and mean (SD) postonset time after stroke of 3 (3.3) years. The range of age is between 20 and 75, and the range of the postonset time after stroke is between 4 months and 16 years.

**TABLE 3:**
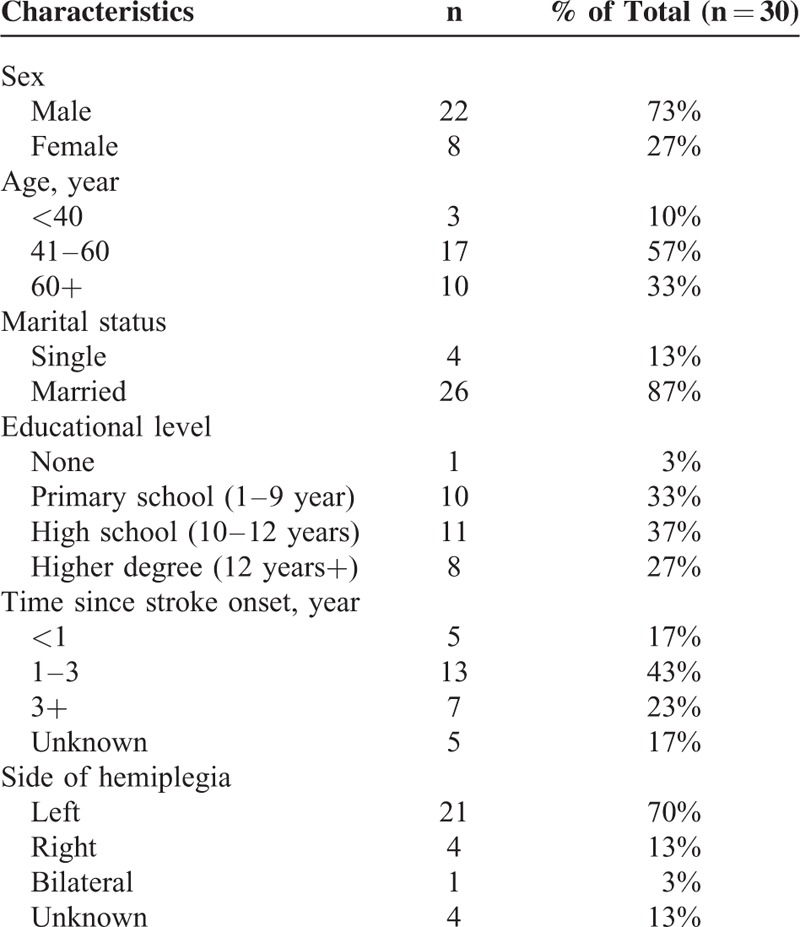
Demographic Characteristics of the Patients

### Clinical Therapists

Nineteen OTs from 2 rehabilitation centers, Shin Kong Wu Ho-Su Memorial Hospital and Taipei Medical University's Shuang-Ho Hospital, participated in rehab-preference survey. Furthermore, 14 OTs out of them participated in rehab-compatibility survey. The demographic and clinical characteristics of the clinical therapists are given in Table 4. The range of age is between 23 and 52, with 2 to 20 years of experience providing neuro-rehabilitation to individuals with stroke.

**TABLE 4:**
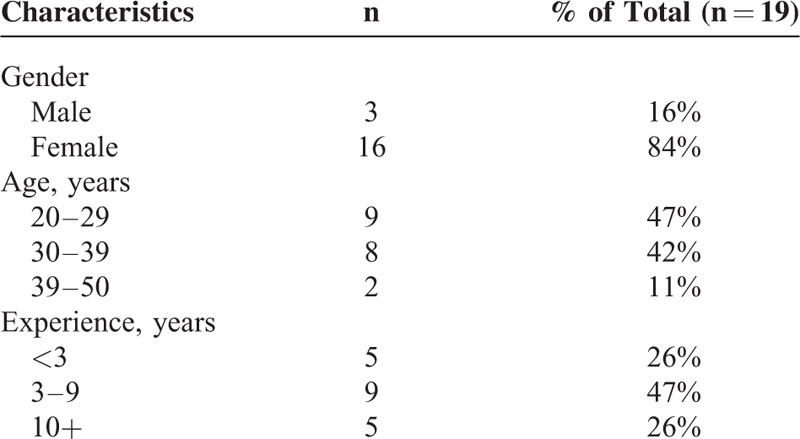
Demographic Characteristics of the Therapists

## RESULTS

### Finding 1: Traditional Rehabilitation Exercises Are Less Effective

**FIGURE 1:**
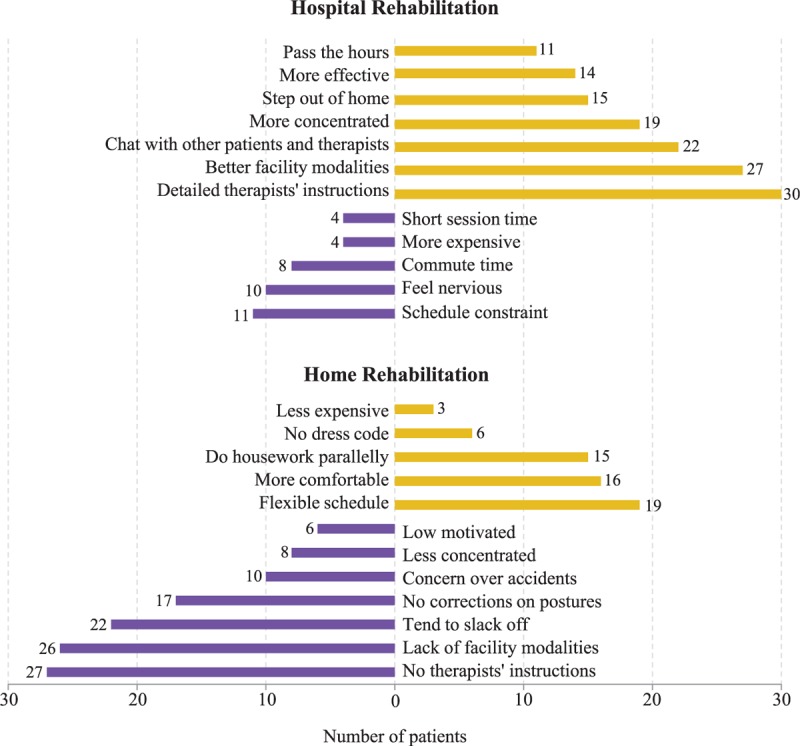
Comparing hospital and home rehabilitation.

All stroke patients perform traditional rehabilitation exercises at home, but they still think performing rehabilitation exercises in the hospital is more effective, although they are aware of the associated overhead (Figure 1). The main drawbacks of home rehabilitation are “No therapists’ instructions” (27), “Lack of facility modalities” (26), “Tend to slack off” (22), and “No corrections on posture” (17). The main advantages of hospital rehabilitation are “Detailed therapists’ instructions” (30), “Better facility modalities” (27), “More concentrated” (19), and “More effective” (14). We believe that this may be partially attributed to the lack of game-based rehabilitation systems at home, which could potentially provide instructions, corrections, and incentives for stroke patients. This also indicates that game-based rehabilitation systems at home should also support remote communication mechanisms such as video conferencing so that therapists can instruct patients remotely. In contrast, the concerned rehabilitation center employs Reha-Slide,^39^ which is a game-based rehabilitation system resulting in more effective rehabilitation.^40^

### Finding 2: Current Game-Based Rehabilitation Systems Lack Entertainment Qualities

The rehabilitation center is equipped with Reha-Slide,^39^ which is an inclinable board with a rod connected to 2 slide-able handles. The rod can be moved in multiple ways, including forward/backward, sideways, and rotation, to facilitate upper extremity functions. Reha-Slide is connected to a desktop computer via a wireless mouse, and the computer runs some games to motivate the stroke patients performing more rehabilitation exercises.

**FIGURE 2:**
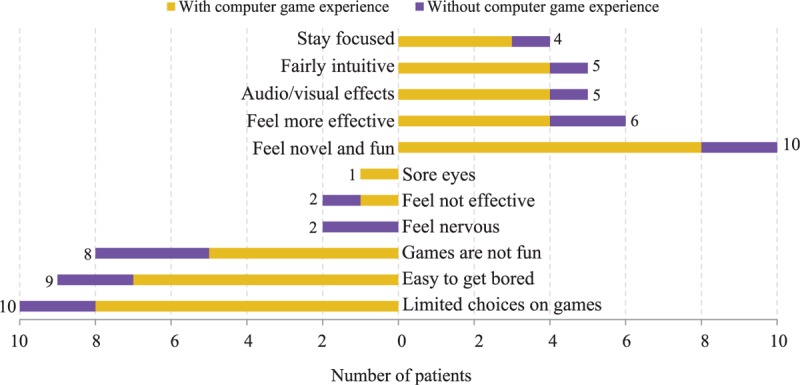
The advantages and disadvantages of Reha-Slide, a game-based rehabilitation system used in the concerned rehabilitation center.

According to our survey, 17 out of 30 stroke patients have rehabilitation experiences on Reha-Slide, and we report only their opinions on the Reha-Slide system below. The subjects are shown to have mixed feelings on existing game-based rehabilitation systems (Figure 2). Although they appreciate the game-based rehabilitation systems, because of, for example, “Feel novel” (10), “Feel more effective” (6), and “Audio/visual effects” (5), they also point out several limitations, for example, “Limited choices on games” (10), “Easy to get bored” (9), and “Games are not fun” (8). Since the top 3 negative opinions are all relevant, if we merge the 3 questions, we will obtain a total of 11 subjects (out of 17, equivalent to 65%) who reported the entertainment and diversity issues of games, especially those who have video gaming experience before. This can be attributed to the fact that the games provided by Reha-Slide are designed by the manufacturer purposefully for rehabilitation and thus they are not as fun as commercial video games and the number of games available on the platform is very limited (only 10 exercises and 3 games). The result, in any case, demonstrates the potentials of game-based rehabilitation systems, although there is room for improvement.

**FIGURE 3:**
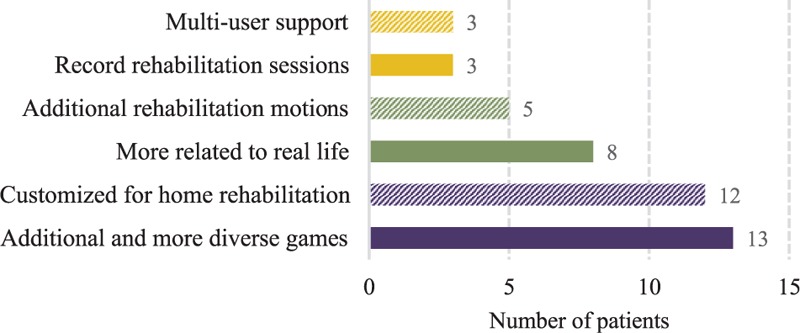
New features for enhancing existing game-based rehabilitation systems.

Next, we ask all the subjects their expectations for the desired game-based rehabilitation systems. They have collectively pointed out several desired new features (Figure 3), such as “Additional and more diverse games” (13), “Customized for home rehabilitation” (12), and “More related to real life” (8). The most desired feature echoes the reported drawbacks of the Reha-Slide system, that is, the diversity of games is critical, as above all, fun should be the most essential driving force to incentivize stroke patients to perform repetitive rehabilitation exercises.

The 2nd-most desired feature is to customize game-based rehabilitation systems for home use. We believe that this implies that such systems should be cost-effective so that they are affordable by most patients; provide remote communications with therapists; and provide social interaction in order to facilitate social support on rehabilitation process.

Meanwhile, the 3rd-most desired feature is to link rehabilitative games to the patients’ personal life experience. For instance, many subjects enjoy Mahjong, Chinese Chess, and card games, while some more outgoing subjects like to hiking, fishing, and playing golf during their leisure time. In addition to the fun element, bringing things that people are passionate about into games is also a common strategy in game design.

To sum up, we make 3 main observations from the above figure:

- patients want to perform rehabilitation exercises with more diverse and fun games;
- patients want cost-effective game-based rehabilitation systems, which are better built on commodity hardware; and
- such systems should take patients’ social interaction needs into account; as we can see, some patients go to hospital simply because of the social support the environment provides.

This indicates that if game-based rehabilitation systems can provide social interactions as though as the patients are in hospital, they would certainly increase the time on such systems when at home.

### Finding 3: Patients Prefer to Use Game-Based Rehabilitation Systems Built Upon Existing Video Games

In the part IV of questionnaire, stroke patients are asked whether the game-based rehabilitation system with existing video games is enjoyable, should it be recommended as part of the rehabilitation program? Exceed 50% of patients state that they would like to use game-based rehabilitation system to rehabilitate but under 25% (most of the patients without video game experience before) respond negative. Internal consistency of the factors was assessed using Cronbach α coefficient. Cronbach α coefficient is 0.89 in the related 5 questions, which suggests a high level of reliability. The results reflect that patients take a positive attitude toward trying game-based systems if they ever played video games.

**FIGURE 4:**
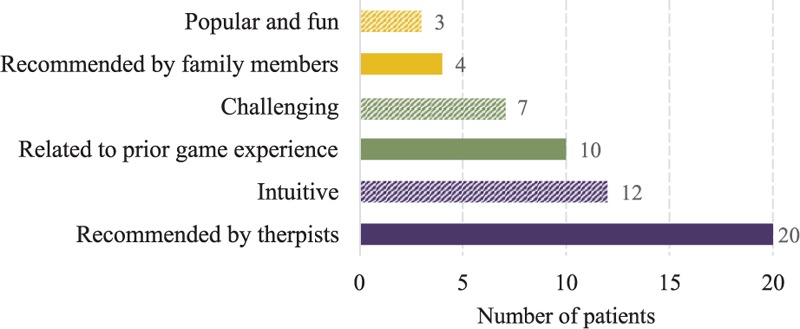
Game selection criteria of stroke patients for rehabilitation systems.

Finally, we ask the patients if they get to choose the games provided by the rehabilitation systems, what are their criteria in choosing the games? The subjects’ reported criteria are depicted in Figure 4. As shown on the graph, the most determinant criterion is “Recommended by therapists” (20). This indicates that patients worry about whether their rehabilitation exercises are right on the track to be effective; thus, therapeutic monitoring and feedback that continuously keeps track of the patients’ exercises and giving therapeutic suggestions and corrections, either locally (by computational intelligence) or remotely (by therapists) would be important features that should seamlessly integrated into game design.

Taking away this criterion, the patients prefer games with the following characteristics: “Intuitive” (12), “Related to prior game experience” (10), “Challenging” (7), and “Popular” (3). We argue that all these criteria are highly correlated to the successfulness of existing designed-for-fun video games. Although these designed-for-rehabilitation games may be beneficial already, most of these games are not designed for entertainments or to be motivational for patients doing rehabilitation. And in aforementioned results, there is still lack of enough available games for patients to select and often difficult to expand their database of game (bad scalability). How to provide and select games that are best suited to individual patients is a big challenge for therapists.

### Finding 4: Patients Prefer to Interact With Game-Based Rehabilitation Systems With Large Displays Using Physical Sensors

**FIGURE 5:**
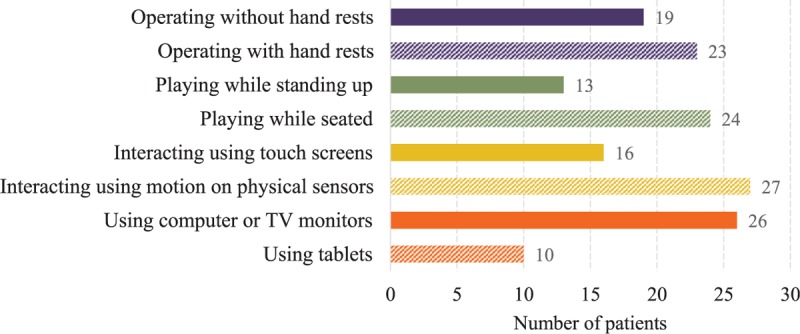
Preferred interaction interfaces of existing video games for stroke patients.

Even though we find out that building game-based rehabilitation systems upon existing video games is attractive, how to perform the conversion is no easy task because most of video games are not designed for stroke patients. Fortunately, our survey reveals some initial insights (Figure 5), for example, more patients prefer to: use larger displays (26), interact with motions (such as Kinect) and physical (such as Wii Remote) sensors (27), and perform rehabilitation exercises while seated (24) with hand rests (23), probably due to their impaired capabilities to stand up and/or raise arms for long-time. These insights, although preliminary, shed some lights on how to develop a platform to convert existing video games to game-based rehabilitation systems. Developing, implementing, and evaluating such a platform is one of our future tasks.

### Finding 5: Therapists’ Experiences Echo Patients’ Opinions

Nineteen OTs with 2 to 20 years of experience, who were providing rehabilitation therapy to patients with stroke, participated in rehab-preference survey. They are all well-experienced to use game-based rehabilitation systems, like Reha-Slide, Bi-Manu-Track,^41^ Hot PluS,^42^ and PAPAMAMA.^43^ In addition, they also have experiences on using off-the-shelf video gaming technologies, such as Wii remote/board and Kinect, for rehabilitation. To know their opinions on current game-based rehabilitation systems, we ask them the following questions which are cited from the questionnaire for patients (QN.I, see in supplementary document) in order to see whether the OTs’ opinions are consistent with the stroke patients’ responses.

The first question is whether these game-based rehabilitation systems should be recommended as a part of the rehabilitation programme? OTs point out the potential usefulness of game-based rehabilitation systems and patients enjoy playing games on these systems. They think that “Feel novel” (15) and “Audio/visual effects” (12) are the top 2 features which can motivate patients to perform rehabilitation and intensify treatment. However, they all agree that there are a number of limitations in current game-based rehabilitation systems, such as “Limited choices on games” (18), “Easy to get bored” (16), “Games are not fun” (13), and “Expensive” (18). The first 3 negative opinions are exactly the same as those given by stroke patients, which indicate that current game-based rehabilitation systems have very limited diversity of games for selection. Moreover, OTs also report that the systems available in hospitals are normally expensive. In our survey, Reha-Slide, Bi-Manu-Track, and Hotplus system cost around US $10,000 to $20,000 dollars. However, patients are only willing to pay US $300 to $1500 dollars for the system. The majority of such systems do not meet cost and deployability requirements for home use, and the total cost of ownership for such systems prevents hospital to provide a variety and a sufficient number of such systems for patient use.

Next, we ask OTs about their desired game-based rehabilitation systems. They report that most of current game-based rehabilitation systems are designed for recovering a specific function, so that these systems cannot be used by a variety of patients. To make a system serviceable for patients at every stage in recovery, OTs have some suggestions to current game-based rehabilitation systems. The most frequently mentioned ones include “more entertainment and diverse games for different ages” (18) and “exercises for more rehabilitation motions” (15). The second-most desired feature is “developing cost-effective systems for home use.” Plus, an ideal system should also include the following functions: “monitoring patients’ rehabilitation and recovery progress,”, “real-time feedback to inform patients about their recovery,” and “report back of patients’ progress to therapists.” These functions are essential for home rehabilitation because patients worry about whether their rehabilitation exercises are correct especially when unsupervised by therapists. Thus, therapeutic monitoring and feedback are important features that should be seamlessly integrated into game design.

Finally, what are the OTs’ criteria in choosing games provided by the rehabilitation systems? Firstly, all the therapists prefer games best suited to specific rehabilitation needs and preferences of the individual patients. For instance, a patient unable to raise arms would be more suitable for games which demand more vertical operations (i.e., games with appropriate tempo and operations) for motor recovery. Secondly, OTs anticipate that the operation motions of playing games are similar to performing conventional facility modalities so that they can easily arrange the categories of the games according to the conventional knowledge and recommend suitable games to patients. Lastly, OTs would prefer games that are “Intuitive” (17), “Popular” (13), and “Challenging” (12). Since the 3 features are all correlated to the successfulness of existing designed-for-fun games, OTs agree commercial games may be a possible solution to provide a variety of games to appeal to different ages, genders, interests, and physical abilities in a cost-effective way. Moreover, if a game play is similar to the prior game experience of the patients, “familiarity” with games (16) was thought to influence patients’ motivation to use these games for rehabilitation.

The above survey shows that OTs’ experiences echo the patients’ responses from the therapeutic viewpoint, which provides solid evidence for our findings. Both of the stroke patients’ and OTs’ feedbacks reveal acceptance of game-based systems as a rehabilitation tool and give a clearer vision for the design of future systems and user interfaces.

### Finding 6: A Simple Rule, Based on Therapists’ Knowledge, of Selecting Commercial Games for Rehabilitation

In rehab-compatibility survey, we obtained ratings of 6 features and 2 overall assessments, compatibility and difficulty, of each game from the questionnaire (QN.II, see in supplementary document). Pearson correlations were used to examine the relation between game features and their difficulties. The confidence level of 0.99 (*P*-value < 0.01) was taken to define statistical significance. The results show that content (*r* = 0.65^∗^), pace (*r* = 0.51^∗^), and number (*r* = 0.47^∗^) are significantly correlated with difficulty. However, other features (distance, range, and size) are not significant. This may be due to the fact that OTs simply used their fingers to play games on a 10? tablet screen, which limited the maximum moving distance of gestures made by fingers; but, these features may be important to other tracking devices such as Kinect, which has broader detecting area to cover whole-body movements. In the overall assessment, compatibility is significantly negatively correlated to difficulty (*r* = −0.53^∗^). Generally, easier games are more compatible with rehabilitation, but without any challenge, patients may lose their motivations to continue game rehab.

**FIGURE 6:**
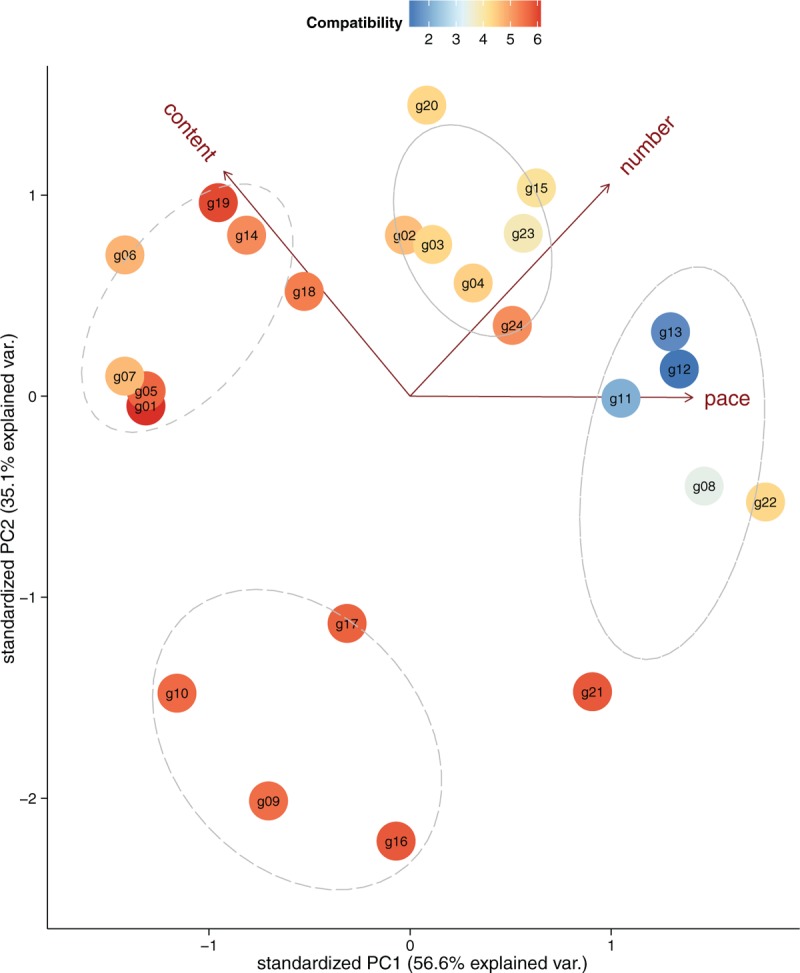
Principle component analysis (PCA) scatterplot showing the clustering pattern of the games in 3 features, content, pace, and number.

We invoke the principle component analysis to map the 3 features, namely content, pace, and number, to 2 principal components, which explain 91.71% of the point variability. The resulting diagram is plotted in Figure 6, where each circle represents a game and its color is the rank of compatibility (derived from the average of OTs’ ratings). The 3 arrows point to each feature, respectively, and most games with similar features are clustered together. There are 4 groups in the plot:

- games which focus on logical and conceptual challenges are ranked higher on content, for example, g01 (Flow Frenzy), g06 (Kids Slide Puzzle), and g19 (Memory Games For Adults);
- games which emphasize hand–eye coordination and reaction-time get ranked higher on pace, for example, g11 (Temple Run), g12 (Subway Surfers), and g13 (Super Laser);
- games with various game objects displayed on the screen get number ranked higher, for example, g02 (Pudding Pop), g03 (Jewels), and g04 (Candy Crush); and
- games different from the other 3 groups are with simple logic content, lower pace, and intuitive interface, for example, g09 (3D Bowling), g10 (Basketball Shoot), and g16 (Kick Zombie).

### The Results Suggest Three Principles for Selecting Rehab-Compatible Games

- Adequate logic challenge: games with adequate logic challenge will attract the interests of patients. Therapists think games emphasizing logic and conceptual challenge (e.g., puzzle games and board games) are attracting to patients whose leisure activities (e.g., Mahjong, Chinese Chess, and card games) have similar features. “Familiarity” with game content was thought to be the main concern for selecting games.
- User-driven: user-driven games (i.e., players take the initiative and the game play is not restricted in time) are much more compatible with rehabilitation; rapid-paced games requiring instant response and fast movements may lead patients to be nervous.
- Intuitive interface: games should be designed with intuitive and simple human–computer interface; relatively complex user interface (with numerous small elements on a display) may lead patients with reduced visual accommodation, such as “neglect” syndrome, to be confused and dazzled during the exercise.

## DISCUSSION

From above results, it is worthy of note that many patients want to perform game-based rehabilitation exercises at home for long-term recovery if they are consistently incentivized and such systems are affordable. Therapists point out the potential usefulness of home systems such as Wii, Kinect, and tablets as hardware platforms for therapy. However, both patients and therapists agree that current game-based rehabilitation systems have limited entertainment and diversity of gaming content, and the cost is also out of personal reach for most people.

Game-based rehabilitation is less likely to be employed for games that are designed from scratch by researchers. Due to the short lifecycle (i.e., getting bored soon) and long development cycle of a game, the supply cannot meet the demand. Rather than developing these games from scratch, the more possible way to provide more games for rehabilitation is adopting existing games. Fortunately, a huge variety of designed-for-fun video games are available at low prices or even free. Today, every Internet user can find and download more than 463,000 iOS games on APP Store^44^ and 128,000 Android games on Google Play.^45^ These games are designed with various motivational features,^34^ such as in-game medals, video playbacks, and bonuses, to encourage the user repetitively to improve game performance and promote opportunities for social connection. However, not all commercial games can be suitable for patients in rehabilitation. Some of these designed for players with full range of motion require fast movements and are therefore too difficult for people with motor impairments to perform.^27^ Nevertheless, as long as 1% of existing video games can be selected out, with the help of the selecting principles we have defined in this study, and incorporated into rehabilitation, more than thousands of games can be used to motivate and engage patients undergoing therapy.

In summary, we believe that selecting games for rehabilitation based on designed-for-fun video games would be a more economic, more feasible, and much more scalable way to provide strong and personalized motivation for various stroke patients, especially those who have video gaming experience before. Therapists can easily assign rehabilitation motions accompanied with corresponding hardware to patients to meet different goals of therapy, and stroke patients can play any rehab-compatibility games they like during rehabilitation. Creating a platform using commodity hardware with existing, appropriately selected games is a promising approach to bring the game-based rehabilitation systems to patients’ home and to increase the patient satisfactions, which leads to lower cost, less commute, and more familiar/private atmosphere. As further study is still required to make arbitrary compositions between rehabilitation motions, software (rehab-compatibility games), and hardware (commercial off-the-shelf tracking devices or conventional facility modalities), we firmly believe that the resulting platform will motivate stroke patients and may also be extended for patients with other disabilities.

### Study Limitations

The current survey study reveals some insights on the requirements of the platform that converts existing designed-for-fun video games to game-based rehabilitation systems for stroke patients. We however agree that a more comprehensive study is needed to guide the detailed designs of such a platform, and that is part of our future work.

## CONCLUSIONS

To the best of our knowledge, our survey is the first of its kind that quantitatively and qualitatively analyzes the stroke patients’ opinions on the problems of current game-based rehabilitation systems and, at the same time, provide new insights of designing future game-based rehabilitation systems.

In this paper, we elaborate the importance of bringing game-based rehabilitation systems home. We show the limitations of existing game-based rehabilitation systems and argue that converting the existing designed-for-fun video games into game-based rehabilitation systems for stroke patients can be more cost-effective. We also point out a few preliminary principles on selecting rehab-compatible games, while the detailed system design, implementation, and evaluations are our future tasks. We envision that the resulting platform will bring more game-based rehabilitation systems to patients’ home, attract more patients to use these systems, and thus be motivational for patients doing rehabilitation.

## Supplementary Material

Supplemental Digital Content
