# Supplementary material for: What Do Stroke Patients Look for in Game-Based Rehabilitation: A Survey Study
Source: Medicine (Baltimore). 2016 Mar 18;95(11):e3032. doi: 10.1097/MD.0000000000003032 (PMC4839901; doi:10.1097/MD.0000000000003032)
Supplement: Supplemental Digital Content [file medi-95-e3032-s001.pdf]

No.：   Date：____/____/______

This is a questionnaire survey aims to understand the current status of rehabilitation of stroke patients, and investigate stroke patients’ expectations and needs to game-based rehabilitation system. Please take the time to give your honest opinion on each of the questions, as this will help us to improve in game-based rehabilitation system. All your answers will be kept in the strictest of confidentiality. Thank you in advance for the help you are giving us.

Institute of Information Science, Academia Sinica
Department of Biomedical Engineering, National Yang-Ming University

I. Demographic Data

The following questions help us to monitor the demographics of our respondents. Naturally, these results will be fully confidential.

1. Gender

☐ Male ☐ Female

2. Age (y)

☐ Below 30 ☐ Between 30 and 40 ☐ Between 40 and 50

☐ Between 50 and 60 ☐ Between 60 and 70 ☐ Between 70 and 80

☐ Above 80

3. Marital status

☐ Single: unmarried, separated, divorced, widowed.

☐ Married: common-law married, living together.

4. Number of children

____________ (Put 0 if no child.)

5. Educational

☐ None ☐ Primary school ☐ High school ☐ Higher degrees

6. Job (or the most recent one)

Please explain: ____

7. Time of stroke onset (y)

Self-reported : _______________/_________________/_______________

Clinical history :_______________/_________________/_______________

8. Site of stroke

☐ Cerebral ☐ Cerebellum ☐ Brainstem

9. Number of strokes

☐ 1st time _____________ (More than 1)

10. Nature of stroke

☐ Ischemic ☐ Hemorrhagic

11. Side of hemiplegia

☐ Left side ☐ Right side ☐ Bilateral

12. Brunnstrom stage (Proximal side of upper limb / Distal side of upper limb / Lower limb)

__________________/_________________/_________________

II. Past hobbies

Please answer the following questions that are related to your leisure activities and video game experience.

A. Please choose your favorite leisure activities. (Check all that apply)

1. Entertainment activity

☐ Watching TV ☐ Singing ☐ Listening to music
☐ Playing puzzle games ☐ Poker games ☐ Gambling games
☐ Chess games
☐ Others (Please explain: ______________________________)

2. Exercise

☐ Ball games ☐ Hiking / Jogging ☐ Boxing
☐ Mountain climbing ☐ Riding bike ☐ Swimming
☐ Dancing ☐ Rope skipping ☐ Ice skating
☐ Yoga ☐ Body-building
☐ Others (Please explain: ______________________________)

3. Skill / Artistry

☐ Calligraphy / Painting ☐ Culinary arts ☐ Handcraft
☐ Flower arrangement ☐ Needlework ☐ Photography
☐ Playing musical instruments
☐ Others (Please explain: ______________________________)

4. Leisure activity

☐ Tea arts ☐ Gardening ☐ Fishing
☐ Raising / Keeping pets
☐ Others (Please explain: ______________________________)

B. Please answer the following questions according to your experience of playing video games

1. Which video game platform have you ever played? (Check all that apply)

☐ Computer games
☐ Arcade games
☐ Console games (Nintendo Wii、Sony PS3、Microsoft Xbox 360)
☐ Hand-held games (smart phone/touch tablet/Play Station Portable)

2. Please select the game types you like. (Check all that apply)

☐ Puzzle game ☐ Sport game ☐ Action Adventure game
☐ Rhythm game ☐ Shooting game ☐ Business simulation game
☐ Others (Please explain: ______________________________)

3. Which one do you like more
   ☐ Single-player game ☐ Multi-Player game

4. On average how often do you play video games per week?
   ☐ Always/ Nearly every day (6-7 days/week)
   ☐ Often (3-5 days/week)
   ☐ Sometimes (1-2 days/week)
   ☐ Seldom/ Hardly ever (under 1 day/week)
   ☐ Never

5. On average how long do you play games each time?
   ☐ Under one hour
   ☐ Between 1 and 3 hours
   ☐ Between 3 and 5 hours
   ☐ Over 5 hours

III. Current rehabilitation status

Please answer the following questions according to current rehabilitation status.

A. Hospital rehabilitation

1. On average how long do you spend on commuting to/from to the hospital?
   ☐ Under half an hour ☐ Between half and one hour
   ☐ Between 1 and 2 hours ☐ Over 2 hours

2. Which rehabilitation program do you enroll at the hospital?
   ☐ Occupational Therapy ☐ Physical Therapy
   ☐ Language Therapy ☐ Psychotherapy
   ☐ Others (Please explain: ____________________)

3. Please check the top three rehabilitation modalities you like. (Check all that apply)
   ☐ None ☐ Sanding box ☐ Standing table
   ☐ Hand pulley ☐ Horizontal towel ☐ Peg board
   ☐ Vertical towel ☐ Stacking cones ☐ Shoulder wheel
   ☐ Wrist wheel ☐ Climbing ladder ☐ Bean bag
   ☐ Reha-Slide ☐ Single curved shoulder arc
   ☐ Others (Please explain: ____________________)

4. Why you like these rehabilitation modalities? (Check all that apply)
   - ☐ Feel novel
   - ☐ Intuitive
   - ☐ Challenging
   - ☐ More effective
   - ☐ Recommended by therapists
   - ☐ Can chat with others
   - ☐ Playing while standing up
   - ☐ Playing while seated
   - ☐ Others (Please explain: ________________________________)

5. Please answer the top three rehabilitation modalities you **do not** like.
   - ☐ None
   - ☐ Sanding box
   - ☐ Standing table
   - ☐ Hand pulley
   - ☐ Horizontal towel
   - ☐ Peg board
   - ☐ Vertical towel
   - ☐ Stacking cones
   - ☐ Shoulder wheel
   - ☐ Wrist wheel
   - ☐ Climbing ladder
   - ☐ Bean bag
   - ☐ Reha-Slide
   - ☐ Single curved shoulder arc
   - ☐ Others (Please explain: ________________________________)

6. Why you **do not** like these rehabilitation modalities? (Check all that apply)
   - ☐ Feel boring
   - ☐ Feel toilsome
   - ☐ Feel not effective
   - ☐ Feel too difficult
   - ☐ Be in disgrace
   - ☐ Cannot chat with others
   - ☐ Others (Please explain: ________________________________)

7. What advantages of performing rehabilitation exercises in the hospital for you? (Check all that apply)
   - ☐ More effective
   - ☐ More concentrated
   - ☐ Detailed therapists' instructions
   - ☐ Better facility modalities
   - ☐ Step out of home
   - ☐ Pass the hours
   - ☐ Chance to chat with other patients and therapists
   - ☐ Others (Please explain: ________________________________)

8. What **disadvantages** of performing rehabilitation exercises in the hospital for you? (Check all that apply)
   - ☐ Feel nervous
   - ☐ Schedule constraint
   - ☐ Commute time
   - ☐ Fear of infectious diseases
   - ☐ Cost
   - ☐ Others (Please explain:________________________________)

B. Home rehabilitation

1. On average how often do you rehabilitate at home per week?
   - ☐ Always (6-7 days/week)
   - ☐ Often (3-5 days/week)
   - ☐ Sometimes (1-2 days/week)
   - ☐ Seldom (under 1 day/week)
   - ☐ Never

2. On average how much time do you spend on rehabilitating each time?
   - ☐ Under half hour
   - ☐ Between half and one hour
   - ☐ Between 1 and 2 hours
   - ☐ Over 2 hours

3. What are the top 3 rehabilitation exercises do you perform at home?
   (1) ____________________
   (2) ____________________
   (3) ____________________

4. What are your living arrangements?
   - ☐ Living alone
   - ☐ Living with caregiver
   - ☐ Living with mate
   - ☐ Living with family
   - ☐ Others (Please explain: ____________________)

5. Relationship with the stroke victim
   - ☐ Mate
   - ☐ Family
   - ☐ Friends
   - ☐ Caregiver
   - ☐ Volunteer
   - ☐ None

6. Where do you perform rehabilitation exercises at home? (Check all that apply)
   - ☐ Living room
   - ☐ Bed room
   - ☐ Balcony/Courtyard
   - ☐ Park
   - ☐ Everywhere
   - ☐ Others (Please explain: ____________________)

7. What are the advantages of performing rehabilitation exercises at home for you? (Check all that apply)
   - ☐ More comfortable
   - ☐ Flexible schedule
   - ☐ No dressing code
   - ☐ Less expensive
   - ☐ Do housework parallelly
   - ☐ Others (Please explain: ____________________)

8. What are the **disadvantages** of performing rehabilitation exercises at home for you? (Check all that apply)
   - ☐ Not motivated
   - ☐ Less concentrated
   - ☐ Tend to slack off
   - ☐ Concern over accidents
   - ☐ No therapists’ instructions
   - ☐ No corrections on postures
   - ☐ Lack of facility modalities
   - ☐ Others (Please explain: ____________________)

IV. Your perception for the past used game-based rehabilitation system

Please answer the following questions according to the experience of using the game-based rehabilitation system (Reha-Slide).

A. What is your perception for the past used game-based rehabilitation system? (Reha-Slide)

1. Do you think using game-based rehabilitation system to rehabilitate was enjoyable for you?

   ☐ Strongly agree ☐ Agree ☐ Neutral
   ☐ Disagree ☐ Strongly disagree

2. What are the advantages of performing rehabilitation exercises by Reha-Slide for you? (Check all that apply)

   ☐ Feel novel ☐ Audio/visual effects
   ☐ Feel more effective ☐ Stay focused
   ☐ Others (Please explain: ________________________________)

3. What are the **disadvantages** of performing rehabilitation exercises by Reha-Slide for you? (Check all that apply)

   ☐ Games are not fun ☐ Easy to get board
   ☐ Limited choices on games ☐ Feel not effective
   ☐ Feel nervous ☐ Sore eyes
   ☐ Others (Please explain: ________________________________)

4. What functions should be added to the system? (Check all that apply)

   ☐ Additional and more diverse games ☐ More related to real life
   ☐ Record rehabilitation sessions ☐ Multi-user support
   ☐ Customized for home rehabilitation
   ☐ Others (Please explain: ________________________________)

B. Please answer the following questions according to your expectations for ideal game rehabilitation systems.

1. Have you ever used any somatosensory game platform? (Check all that apply)

   ☐ Nintendo Wii (Wii Remote) ☐ Robot-aided
   ☐ Microsoft Xbox360 (Microsoft Kinect) ☐ Sensor based
   ☐ Sony PlayStation 3 (PlayStation®Eye 、PlayStation®Move)
   ☐ Others (Please explain: ________________________________)

2. Where have you used somatosensory game platforms? (Check all that apply)
   ☐ None ☐ Hospital ☐ Home
   ☐ Store ☐ Others(Please explain: ____________________)

3. Do you want to perform rehabilitation by using somatosensory games?
   ☐ Strongly agree ☐ Agree ☐ Neutral
   ☐ Disagree ☐ Strongly disagree

4. Where do you want to use this system in addition to at the hospital? (Check all that apply)
   ☐ Living room ☐ Bed room ☐ Balcony/Patio
   ☐ Park ☐ Everywhere
   ☐ Others (Please explain: ____)

5. What are your favorite game modes? (Check all that apply)
   ☐ Single-player mode ☐ Multi-player mode
   ☐ Multi-player mode over the network

6. What are your favorite operations? (Check all that apply)
   ☐ Interacting using motions of physical sensors
   ☐ Interacting using touch screens ☐ Playing while being seated
   ☐ Playing while standing up ☐ Operating with hand rests
   ☐ Operating without hand rests
   ☐ Others (Please explain: ____)

7. What are your favorite display interfaces? (Check all that apply)
   ☐ Using tablets ☐ Using monitors
   ☐ Others (Please explain: ____)

8. Will adding you favorite leisure activities to the rehabilitee system motivate you to try it?
   ☐ Strongly agree ☐ Agree ☐ Neutral
   ☐ Disagree ☐ Strongly disagree

9. What are the top 3 leisure activities do you want to integrate with the rehabilitation system?
   (1) ____
   (2) ____
   (3) ____

10. Have you ever used smart phones or tablets?
   ☐ Yes ☐ No

11. Do you want to play popular games on smart phones or tablets?

☐ Strongly agree ☐ Agree ☐ Neutral
☐ Disagree ☐ Strongly disagree

12. Will integrating the popular games with the rehabilitation system, motivate you to try it?

☐ Strongly agree ☐ Agree ☐ Neutral
☐ Disagree ☐ Strongly disagree

13. Which features do you consider to be the most important in choosing games for rehabilitation? (Check all that apply)

☐ Popular and fun ☐ Intuitive
☐ Challenging ☐ Recommended by therapists
☐ Related to prior game experience ☐ Recommended by family members
☐ Others (Please explain: ____)

14. If budget is not a concern, how much will you spend on the game-based rehabilitation system?

☐ Under NT 5000 dollars ☐ Under NT 10000 dollars
☐ Under NT 50000 dollars ☐ Over NT 50000 dollars
